# The Complete Genome Sequence of *Morelia viridis*, the Green Tree Python

**DOI:** 10.56179/001c.66204

**Published:** 2022-12-27

**Authors:** Benson H. Morrill, Ian S. Bessell, Stacy Pirro

**Affiliations:** 1 Rare Genetics Inc,; 2 S&J Reptiles,; 3 Biodiversity, Iridian Genomes

**Keywords:** genome, snake, python

## Abstract

The Green Tree Python (*Morelia viridis*) is a snake native to New Guinea, some islands in Indonesia, and the Cape York Peninsula in Australia. We present the whole genome sequence for this species. Illumina sequencing was performed on a genetic sample from a single individual. The reads were assembled using a *de novo* method followed by a series of references from related species for finishing. The raw and assembled data are publicly available via Genbank: Sequence Read Archive (SRR19167500) and genome (JANHOE000000000).

## Introduction

The Green Tree Python (Morelia viridis) is a snake native to New Guinea, some islands in Indonesia, and the Cape York Peninsula in Australia. This species is bright green and can reach a total length (including tail) of 2 m (6.6 ft) and a weight of 1.6 kg (3.5 lb), with females slightly larger and heavier than males. Living primarily in trees, the green tree python hunts and eats small reptiles and mammals. It is a popular pet, and numbers in the wild have suffered with large-scale smuggling of wild-caught green tree pythons in Indonesia. Despite this, the green tree python is rated as least concern on the IUCN Red List of endangered species. We present the whole genome sequence for this species.

## Methods

The genetic sample was obtained from a single individual snake that was legally imported by S&J Reptiles in 2015 from the Aru islands and derived from a naturally shed skin.

DNA extraction was performed using the Qiagen DNAeasy genomic extraction kit using the standard process. A paired-end sequencing library was constructed using the Illumina TruSeq kit according to the manufacturer’s instructions. The library was sequenced on an Illumina Hi-Seq platform in paired-end, 2 × 150bp format. The resulting fastq files were trimmed of adapter/primer sequence and low-quality regions with Trimmomatic v0.33 (Bolger, Lohse, and Usadel 2014). The trimmed sequence was assembled by SPAdes v2.5 (Bankevich et al. 2012) followed by a finishing step using Zanfona (Kieras, O’Neill, and Pirro 2021).

## Results

The genome assembly yielded a total sequence length of 1,351,159,366 bp over 110,412 scaffolds.

## Data Availability

Raw and assembled data is publicly available via GenBank: Raw genome data https://trace.ncbi.nlm.nih.gov/Traces/sra/?run=SRR19167500 Assembled genome https://www.ncbi.nlm.nih.gov/nucleotide/JANHOE000000000

## References

[R1] Bankevich Anton, Nurk Sergey, Antipov Dmitry, Gurevich Alexey A., Dvorkin Mikhail, Kulikov Alexander S., Lesin Valery M., 2012. “SPAdes: A New Genome Assembly Algorithm and Its Applications to Single-Cell Sequencing.” Journal of Computational Biology 19 (5): 455–77. 10.1089/cmb.2012.0021. 22506599 PMC3342519

[R2] Bolger Anthony M., Lohse Marc, and Usadel Bjoern. 2014. “Trimmomatic: A Flexible Trimmer for Illumina Sequence Data.” Bioinformatics 30 (15): 2114–20. 10.1093/bioinformatics/btu170. 24695404 PMC4103590

[R3] Kieras M, O’Neill K, and Pirro S. 2021. “Zanfona, a Genome Assembly Finishing Tool for Paired-End Illumina Reads.” https://github.com/zanfona734/zanfona.

